# TCR Gene Transfer: MAGE-C2/HLA-A2 and MAGE-A3/HLA-DP4 Epitopes as Melanoma-Specific Immune Targets

**DOI:** 10.1155/2012/586314

**Published:** 2012-02-12

**Authors:** Trudy Straetemans, Mandy van Brakel, Sabine van Steenbergen, Marieke Broertjes, Joost Drexhage, Joost Hegmans, Bart N. Lambrecht, Cor Lamers, Pierre van Der Bruggen, Pierre G. Coulie, Reno Debets

**Affiliations:** ^1^Laboratory of Experimental Tumor Immunology, Department of Medical Oncology, Erasmus MC, 3015 GE, Rotterdam, The Netherlands; ^2^Department of Pulmonary Diseases, Erasmus MC, 3015 GE, Rotterdam, The Netherlands; ^3^Ludwig Institute for Cancer Research Ltd, Brussels Branch, and de Duve Institute, Université Catholique de Louvain, 1200 Brussels, Belgium

## Abstract

Adoptive therapy with TCR gene-engineered T cells provides an attractive and feasible treatment option for cancer patients. Further development of TCR gene therapy requires the implementation of T-cell target epitopes that prevent “on-target” reactivity towards healthy tissues and at the same time direct a clinically effective response towards tumor tissues. Candidate epitopes that meet these criteria are MAGE-C2_336-344_/HLA-A2 (MC2/A2) and MAGE-A3_243-258_/HLA-DP4 (MA3/DP4). We molecularly characterized TCR**αβ** genes of an MC2/A2-specific CD8 and MA3/DP4-specific CD4 T-cell clone derived from melanoma patients who responded clinically to MAGE vaccination. We identified MC2/A2 and MA3/DP4-specific TCR-V**α**3/V**β**28 and TCR-V**α**38/V**β**2 chains and validated these TCRs *in vitro* upon gene transfer into primary human T cells. The MC2 and MA3 TCR were surface-expressed and mediated CD8 T-cell functions towards melanoma cell lines and CD4 T-cell functions towards dendritic cells, respectively. We intend to start testing these MAGE-specific TCRs in phase I clinical trial.

## 1. Introduction

Adoptive therapy with antigen-specific T cells has shown clinical successes in the treatment of viral infections and tumors [1–5]. Receptor gene therapy, in which patients are treated with gene-engineered T cells with either chimeric antigen receptors (CARs) or T-cell receptors (TCRs), provides an attractive alternative to provide therapeutic immunity. Clinical application of gene-engineered T cells to treat various tumor types, such as renal cell cancer, ovarian cancer, neuroblastoma, lymphoma, melanoma, and colorectal and synovial cancers proved feasible but, despite some successes, generally did not show antitumour responses in a substantial number of patients [6–13]. Notably, in an early clinical trial to treat metastatic renal cell cancer with CAR-engineered T cells, with total T-cell doses as low as 2 × 10^8^ T cells, we observed reversible yet discrete cholangitis and damage to bile duct epithelium as a likely consequence of T-cell localization and expression of the target epitope carbonic anhydrase IX (CAIX) on normal tissue [6]. Subsequent trials with CARs directed against Her2/Neu and CD19 and TCRs directed against the HLA-A2-restricted antigens MARTI, gp100 and CEA, have confirmed this notion [11, 12, 14, 15]. Collectively, these studies underscore the need for T-cell target epitopes that are expressed on malignant tissue in a highly restricted manner and are able to initiate a clinically effective T-cell response. 

Cancer testis antigens (CTAs) are immunogenic proteins expressed in many tumors but silenced in normal cells except for male germline cells, placenta, and thymic medullary epithelial cells [16, 17]. *In vitro* studies have provided initial proof that gene transfer of TCR*αβ* directed against MAGE-A1/HLA-A1, MAGE-A3/HLA-A2, and NY-ESO-1/HLA-A2 as well as NY-ESO-1/HLA-DP4 result in effective and CTA-specific T-cell responses [18–21]. Of the group of CTA, in particular the MAGE antigens constitute attractive candidates for immune therapy giving not only tumour-specific expression but also their role in tumour biology, expression in multiple tumours, and potential to constitute effective T-cell targets. Four families of MAGE genes are located on chromosome X: *MAGE-A (12 genes), B (6 genes)*, *C (4 genes), and D (2 genes)*. Up to now, there are over 50 identified combinations of MAGE peptides and HLA class I or class II molecules, recognized by CD8 or CD4 T cells, respectively (see for an overview: http://cancerimmunity.org/peptidedatabase/Tcellepitopes.htm). We propose the MAGE-C2_336–344_/HLA-A2 (MC2/A2) peptide ALKVDVEERV and MAGE-A3_243–258_/HLA-DP4 (MA3/DP4) peptide KKLLTQHFVQENYLEY as candidate T-cell targets for the following reasons. *First*, MC2 and MA3 proteins actively contribute to the development of malignancies. MC2 suppresses p53-dependent apoptosis, thus prolonging tumor survival [22, 23], whereas MA3 mediates fibronectin-controlled progression and metastasis [24], and is expressed by melanoma stem cells [25, 26]. *Second*, MC2 and MA3 are expressed in multiple tumor types and their expression is associated with poor clinical outcome in these tumor types [27–32]. MC2 is expressed in 43% of metastatic melanomas, 33% of head and neck squamous cell cancers, 30% of bladder cancers, and 10% of nonsmall cell lung cancers [28]. MA3 is expressed in 76% of metastatic melanomas [27], in up to 50% of nonsmall cell lung cancer [29], and in many other tumor types such as colon rectal, hepatocyte cellular, prostate and breast cancers, and haematological malignancies such as multiple myeloma [30, 33–36]. Furthermore, HLA-A2 and HLA-DP4 are the most frequent MHC class I and II alleles among Caucasians, that is, representing 44 and 75% of the general population, respectively. And *third*, MC2 and MA3 potentially constitute clinically effective T-cell target epitopes, as evidenced by induction of enhanced numbers of anti-MAGE T cells that paralleled significant and durable clinical responses [37, 38].

The clinical potential of MC2-specific T cells is exemplified by a high frequency of MC2/A2-specific CTL (10^−4^ of CD8 T cells) observed in the blood of a melanoma patient whose tumors regressed after vaccination with MAGE-A1 and A3 peptides, whereas in the same patient the frequency of anti-vaccine CTLs was low (3 × 10^−6^ of CD8 T cells) [37]. A CTL clone recognizing this epitope (EB81-CTL16) was isolated demonstrating the most pronounced increase in frequency not only in blood but also in a regressing cutaneous metastasis (>100 and 1000-fold, resp.). Interestingly, the same patient also showed increased frequencies in blood and a regressing metastasis (up to 200-fold) of other T-cell clones-specific for the same and other MC2 epitopes [39]. In a second melanoma patient who showed tumor regression upon MAGE vaccination, the most frequent antitumor CTL clone was again directed against a MC2 epitope [40]. With respect to MA3, various trials have been performed substantiating its clinical potential as a T-cell target. A phase II clinical trial with highly purified MA3 protein in nonsmall cell lung cancer showed a significant reduction in relative risk of cancer recurrence following surgery in vaccinated versus placebo-treated patients [41]. This MA3 vaccine provided B-cell responses, CD8 T-cell responses as well as HLA-DP4-restricted CD4 T-cell responses against the MA3 KKL epitope in lung cancer patients [42, 43]. Recently, a phase III trial started to investigate the efficacy of MA3 antigen vaccination after tumor resection in lung cancer patients [44]. Also in melanoma patients, MA3 protein vaccinations using either protein or MA3-expressing PBMC initiate antigen-specific immune responses [45]. Vaccinations with dendritic cells loaded with MA3/DP4 peptide rapidly induced peptide-specific T-helper-cell responses in melanoma patients. Median survival in vaccinated patients was longer than in untreated control patients and showed no signs of major toxicities due to vaccination [46] and personal communication (Gerold Schuler, Erlangen, Germany).

In this study, we chose MC2_336–344_/A2 and MA3_243–258_/DP4 as T-cell epitopes, and cloned and characterized the corresponding TCR*αβ* genes of CD8 and CD4 T-cell clones derived from two metastatic melanoma patients who responded clinically to MAGE-vaccination. TCR*αβ* genes were then introduced into primary human T cells, and tested for surface expression and MAGE-specific CD8 and CD4 T-cell functions *in vitro*.

## 2. Materials and Methods

### 2.1. Melanoma Patients EB81 and R12 and Patient-Derived T-Cell Clones

In a vaccination study, metastatic melanoma patient EB81 received cutaneous vaccinations with recombinant canarypox (ALVAC) virus, carrying a minigene encoding antigenic MAGE-A1 and A3 peptides that are presented by HLA-A1. These were followed by vaccinations with the same peptides. One year after the first vaccination, all cutaneous metastases had disappeared, and the patient remained tumor-free for 3 years [37]. CTL-606C/22.2 (EB81-CTL 16) is a cytotoxic CD8 T-cell clone derived from EB81 whose increase in frequency is most pronounced in various body compartments upon vaccination with MAGE, and it recognizes MC2_336–344_/HLA-A2 (ALKVDVEERV) [37]. Melanoma patient 12 was included in a clinical trial in which mature monocyte-derived dendritic cells loaded with multi-HLA class I and II peptides (including MAGE-A3_243–258_ peptide) were administered subcutaneously [38]. CD4 T-cell clone R12-C9, recognizing MA3_243–258_/HLA-DP4 (KKLLTQHFVQENYLEY), was derived from PBMC from melanoma patient 12, after *in vitro* stimulation with MA3_243–258_/DP4 peptide and sorted on IFN*γ* secreting CD4+ T cells by FACSVantage flow cytometer (BD Biosciences) as described earlier [46]. CTL clones 16 and R12-C9 were cultured in IMDM with 10% human serum, glutamine, and antibiotics.

### 2.2. Other Cells and General Reagents

PBMC from healthy donors were isolated by centrifugation through Ficoll-Isopaque (density = 1.077 g/cm^3^; Amersham Pharmacia Biotech, Uppsala, Sweden). Transduced primary human T cells were cultured in RPMI 1640 medium supplemented with 25 mM HEPES, 200 mM L-glutamine, 10% human serum, antibiotics, and 360 IU/mL human rIL-2 (Proleukin; Chiron, Amsterdam, The Netherlands) and stimulated every 2 weeks with a mixture of irradiated allogeneic feeder cells as described elsewhere [47]. The human embryonic kidney cell line 293T and Phoenix-Ampho, both used to package retroviruses carrying RNA encoding TCR*αβ*, were grown in DMEM with 10% fetal bovine serum (FBS; Greiner Bio-one Alphen a/d Rijn, The Netherlands), glutamine, antibiotics, and 1% MEM nonessential amino acids. The same medium plus supplements was used to grow the melanoma cell lines EB-81-MEL-2 (MC2/A2^pos^) and MZ2-MEL43 (MA3/DP4^pos^). An MC2^neg^/A2^pos^ and MA3^neg^/DP4^pos^ B lymphoblast cell line (BSM) and an EBV transformed HLA-DP4^pos^ B cell line (i.e., EBV-MAGJ) transduced with retrovirus encoding li-MA3 cDNA as described in [48] (i.e., EBV-MA3) were cultured in RPMI supplemented with glutamine, antibiotics, and 10% FBS. The melanoma cell line EB-81-MEL-2 and the B cell line EBV-MAGJ were derived from the same patients from whom the T-cell clones were derived (melanoma patient EB-81 and patient 12, resp.). In some cases, target cells were pretreated with 50 pg/mL human recombinant IFN*γ* (Peprotech, Rocky Hill, NJ, USA) for 48 h prior to functional T-cell assays.

MC2/A2 peptide MHC (pMHC) complexes were ordered from Proimmune (Oxford, UK). MA3/DP4 pMHC complexes were produced in S2-drosophila insect cells, essentially as described previously [46]. We used the following mAbs: anti-CD4 (clone 13 B8.2, BD Biosciences, Erembodegem, Belgium), anti-CD8 (clone SK1, BD Biosciences) and anti-TCR-V*β*2 mAbs (clone MPB 2D5, Immunotech, Marseille, France). Other reagents used were the HLA-A2-binding peptides MC2_336–344_ (ALKVDVEERV) and (as a control) gp100_280–288_ (YLEPGPVTA), the HLA-DP4-binding peptide MA3_243–258_ (KKLLTQHFVQENYLEY) (all three from Eurogentec, Maastricht, The Netherlands), Phytohemagglutinin (PHA) (Remel Ltd, Lenexa, KS, USA), Phorbol 12-Myristate 13-Acetate (PMA) (Sigma-Aldrich, St. Louis, MO, USA), GM-CSF, IL-4, TNF*α* (all three from PeproTech) and PGE2 (Sigma-Aldrich).

### 2.3. MAGE-A3 Protein

MA3 protein was expressed by the Des insect cell expression system (Invitrogen, Breda, The Netherlands). To this end, MA3 cDNA was cloned into the pMT/BiP/V5-His vector and, together with the pCoHygro vector, introduced into S2-insect cells by nucleofection (Amaxa Biosystems, Cologne, Germany) according to the manufacturer's guidelines. MA3 protein expression by transfected S2 cells, at a density of 3 × 10^6^/mL, was induced by copper sulfate (500 *μ*M). Five days after induction of protein expression, culture medium was harvested and soluble MA3 protein was purified by FPLC (Acta, GE Healthcare, Zeist, The Netherlands) using a histrap column, followed by size exclusion on a sephadex 75 column.

### 2.4. Genes Encoding TCR*αβ*-Specific for MC2/A2 and MA3/DP4

**Figure 1:**
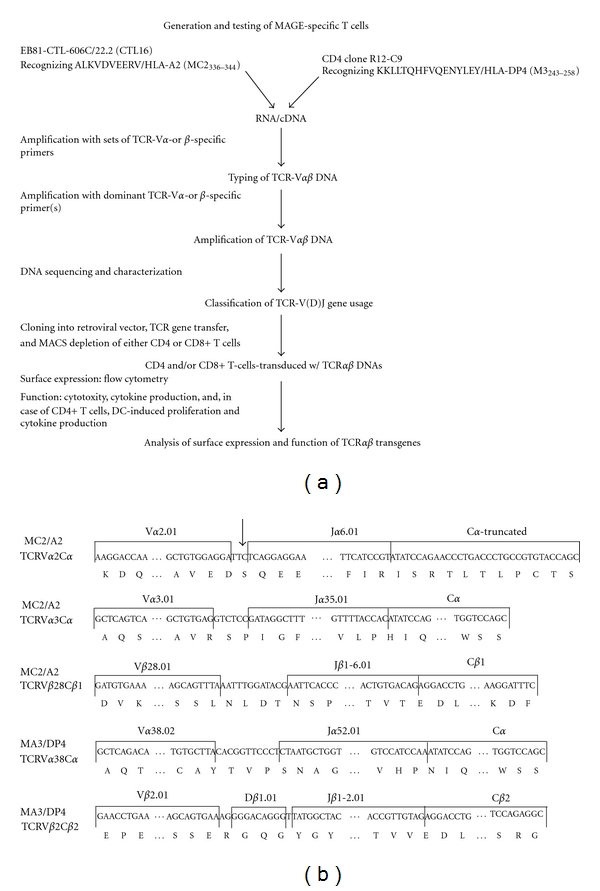
Cloning and validation of MC2/A2 and MA3/DP4 TCR*αβ* genes. (a) Schematic representation of how TCR DNAs have been cloned, typed for TCR-V(D)J gene usage, and tested in T cells following gene transfer. (b) TCR-V(D)J and C classification of the TCR*α* and *β* chains expressed by EB81-CTL16 and R12-C9 according to http://www.imgt.org/. The arrow before the J*α*6.01 indicates a frame shift preventing surface expression of this TCR-V*α*2 chain. Sequence data for human TCR-V*α*2, V*α*3, and V*β*28 of EB81-CTL16-derived TCR genes are available from GenBank under accession nos. EU427373, EU427374, and EU427375, respectively; and sequence data for human TCR-V*α*38 and V*β*22 of R12-C9-derived TCR genes are available from GenBank under accession nos. EU427376 and EU427377, respectively.

RNA was isolated from T-cell clones EB81-CTL16 and R12-C9 and reverseLy transcribed with Superscript III (Invitrogen) according to the manufacturer's instructions. The TCR-V*α* and V*β* regions were amplified and family-typed using a set of sense primers, covering all variable segments, in combination with either a TCR-C*α* or C*β* antisense consensus primer. Nested PCR was performed on TCR-V*α* and V*β* products before gel electrophoresis. Primers specific for C*β*1 or C*β*2 were used to discriminate between both C*β* genes. Positive PCR products were cloned, and plasmid DNAs from at least 5 independent colonies were sequenced. Specific primers were then used to amplify full-length (FL) TCR*α* and *β* DNAs from CTL-derived cDNA. In some cases, (i.e., MA3/DP4 TCR*β*) primers were also used to amplify control TCR DNAs from a spleen cDNA library. Standard primers were used to amplify the TCR*α* and TCR*β* DNAs and will be provided upon request. TCR*α* and *β* genes were cloned as wild-type TCRs into two separate pBullet retroviral vectors [49] (abbreviated as pB: TCR*α*+*β*) or as codon-optimized TCRs in a TCR*β*-2A-TCR*α* cassette in a single pMP71 vector (abbreviated as pMP71: optTCR*β*-2A-TCR*α*, see *Supplementary text and figures available online at doi: 10.1155/2012/586314*). The strategy we employed to clone MAGE-specific TCR*αβ* genes and to test their surface expression and function following TCR gene transfer is depicted in Figure 1(a).

### 2.5. Transduction of Human T Lymphocytes

Human T lymphocytes of healthy donors were activated with anti-CD3 mAb and transduced with retrovirus harboring either MAGE-specific or control TCR*α* and *β* transgenes. The transduction procedure was described by Lamers and colleagues [50] except that in the current study TCR-encoding retroviruses were produced by a coculture of 293T and Phoenix-Ampho packaging cells. T cells were FACSorted using the corresponding p/MHC multimer prior to functional assays. For some experiments, the MA3/DP4 TCR transduced T cells were depleted either for CD4 or CD8 T cells using anti-CD4 or CD8 mAb-coated and PE-labeled magnetic beads and MACS columns (Miltenyi, Bergisch Gladbach, Germany) according to the manufacturer's instructions.

### 2.6. Flow Cytometry of TCR-Transduced T Lymphocytes

MC2/A2 TCR-transduced T cells were analyzed for TCR expression by flow cytometry using PE-labeled MC2/A2 pentamers (10 nM). MA3/DP4 TCR-transduced T cells were analyzed for TCR expression by PE-labeled anti-TCR-V*β*2 mAb, PE-labeled MA3/DP4 tetramers (50 nM), and anti-CD4 mAb. For immunostaining, 0.5 × 10^6^ transduced T cells were washed with PBS and incubated with MC2/A2 pentamer (or antibodies) at 4°C for 30 min or with MA3/DP4 tetramer at 37°C for 2 h. Upon completion of the immunostainings, cells were washed and fixed with 1% paraformaldehyde. Events were acquired and analyzed on a Cytomics FC 500 flow cytometer with CXP software (Beckman Coulter, Mijdrecht, The Netherlands).

### 2.7. Cytotoxicity Assay

Cytotoxic activity of T cells was measured in a standard 6 h ^51^Cr-release assay as described previously [51]. Peptide loading of target cells was performed by addition of either MC2, gp100 (control) or MA3 peptide (final concentrations at 10 *μ*M) for 15 min at 37°C and 5% CO_2_ prior to cocultivation with effector T cells.

### 2.8. Cytokine Production

To quantify the production of cytokines after antigen-specific stimulation, 6 × 10^6^ T cells were cultured in the presence of 2 × 10^6^ target cells for 18 h at 37°C and 5% CO_2_. As a positive control, T-cell transductants were stimulated with PHA and PMA. Supernatants were harvested, and levels of IFN-*γ* and TNF-*α* were determined by standard ELISA (Sanquin, Amsterdam, The Netherlands).

### 2.9. CD4 T-Cell Assay

CD4 T-cell assays were based on dendritic cell: CD4 T-cell cocultures. To generate autologous dendritic cells (DCs), we used PBMC from the same HLA-DP4-positive donor that had been used to generate MA3/DP4 TCR-transduced CD4 T cells. PBMC were MACS-enriched using CD14 microbeads (Miltenyi Biotech), seeded at 10^6^ cells/mL in RPMI 1640 medium without HEPES and supplemented with glutamine, 10% FBS, 10 *μ*g/mL gentamycine, and the cytokines GM-CSF (1000 IU/mL) and IL-4 (200 IU/mL). At day 6, cells were used as a source of immature DC and incubated with MA3 protein (25 *μ*g/mL) either in the absence or presence of TNF*α* (200 IU/mL) and PGE2 (5 *μ*M) for an additional 2 days resulting in immature or mature MA3-positive DC, respectively. DC maturation state was confirmed by flow cytometric analysis of surface expression of CD80, CD86, and HLA-DR.

Immature or mature MA3-positive DCs were washed and added at 2 × 10^4^ per round-bottomed microwell to 2 × 10^5^ CD4 T cells in 200 *μ*l T-cell medium. After 4 days of DC: T-cell coculture, supernatants were harvested, and cytokine production was determined in culture supernatants with Cytokine Bead Array (Th1/Th2 CBA kit, BD Biosciences) according to the manufacturer's instructions.

## 3. Results

### 3.1. Sequences of TCR*αβ* Genes from MAGE-Specific T-Cell Clones

**Figure 2:**
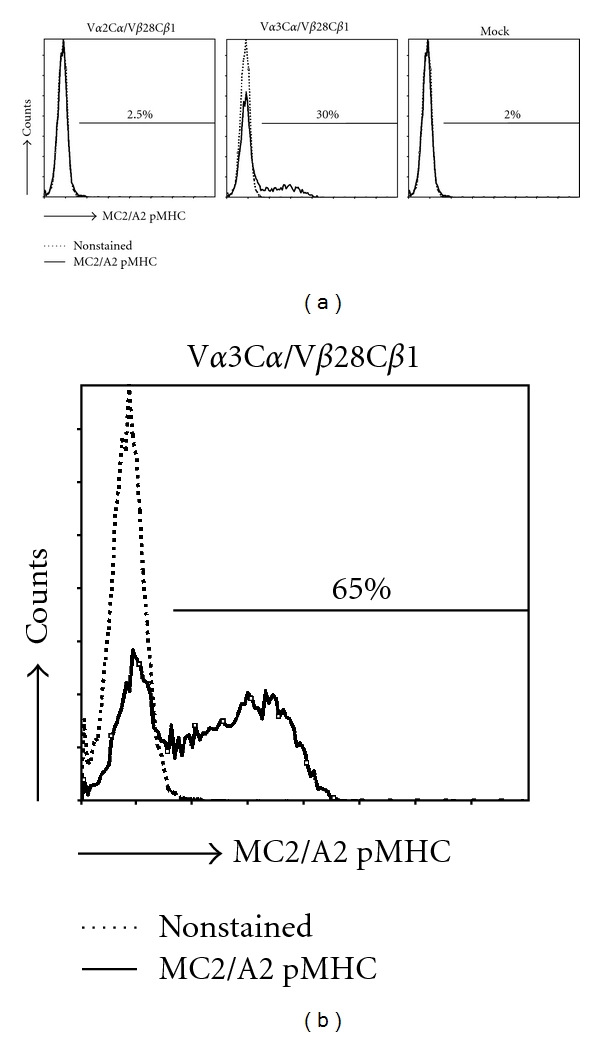
Primary human T cells transduced with TCR-V*α*3/V*β*28 genes bind MC2/A2 pMHC. The MC2/A2 TCR T cells were labeled with PE-conjugated MC2_336–344_/A2 pentamers before flow cytometric analysis (solid lines). (a) T cells transduced either with TCR-V*α*2C*α*/V*β*28C*β*1 and V*α*3C*α*/V*β*28C*β*1 or control TCR*αβ* genes (Mock), and not sorted for MC2/A2 binding. (b) T cells transduced with TCR-V*α*3C*α*/V*β*28C*β*1 genes and FAC Sorted with MC2/A2 pentamer. Results are from a representative transduction out of 6 transductions of PBMC from 2 donors with similar results.

CD8 T-cell clone EB81-CTL16 and CD4 T-cell clone R12-C9, which were established from melanoma patients following MAGE vaccinations, were used to obtain genes encoding for MC2/A2- and MA3/DP4-specific TCR*αβ*'s. Sequence characterization revealed that EB81-CTL 16 harbored genes encoding TCR-V*α*2.01/J*α*6.01/C*α*, V*α*3.01/J*α*35.01/C*α*, and V*β*28.01/J*β*1-6.01/C*β*1, whereas R12-C9 harbored genes encoding TCR-V*α*38.02/J*α*52.01/C*α* and V*β*2.01/D*β*1.01/J*β*1-2.01/C*β*2. We found that the TCR-V*α*2.01/J*α*6.01/C*α* contained a frame shift in the J*α* region (Figure 1(b)). As a result, there was a premature stop codon in the constant domain and no surface expression of this TCR*α* chain (see Figure 2(a)). Figure 1(b) shows the exact nucleotide and amino acid sequences of the various MAGE TCR chains and their corresponding TCR-V(D)J and C classifications (according to http://www.imgt.org/).

### 3.2. TCR-V*α*3/V*β*28 Chains Confer T Cells with the Ability to Bind MC2_336–344_/A2 Ligands

Retroviral transduction of human primary T cells with the TCR-V*α*3C*α* and V*β*28C*β*1 chains but not with irrelevant TCR*α* and *β* chains (i.e., mock TCR) resulted in TCR surface expression and binding to multimers of recombinant HLA-A2 molecules folded with MC2_336–344_ peptide (Figure 2(a)). Enrichment of TCR-transduced T cells (TCR T cells) with MC2/A2 multimers by FACSort resulted in higher proportions of T cells expressing the MC2/A2 TCR (30% versus 65% pMHC binding before and after sort, Figure 2(b)). TCR surface expression was stable for at least three months (data not shown).

### 3.3. TCR-V*α*3/V*β*28-Transduced Primary Human T Cells Show Antigen-Specific Functions *In Vitro*

**Figure 3:**
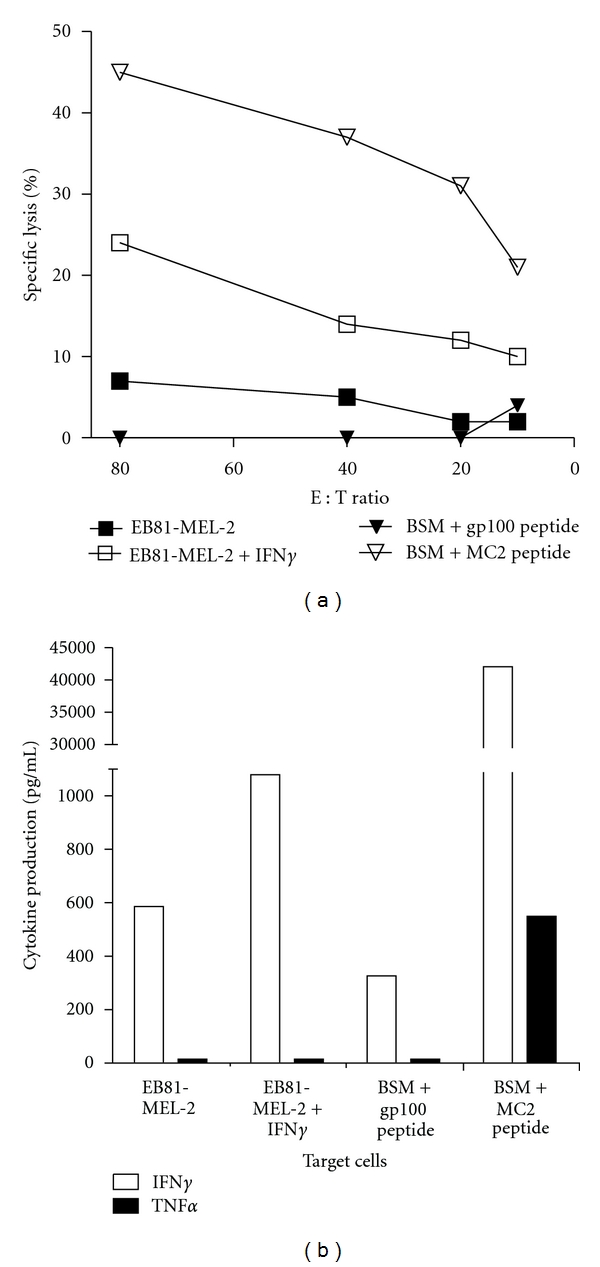
MC2/A2 TCR is functionally expressed by primary human T cells. (a) MC2/A2 TCR T cells lyse MC2/A2 positive target cells. TCR T cells were tested in a 6 h ^51^Cr-release assay. The following target cells were used: MC2/A2-positive EB81-MEL-2 melanoma cells (derived from the same patient from whom the MC2 TCR was derived), pretreated or not with IFN*γ*, and A2-positive BSM EBV-B cells, pulsed either with gp100 or MC2 peptide (both at 10 *μ*M final). Mock T cells did not lyse MC2/A2-positive target cells (data not shown). Effector-to-target cell ratios are indicated on the *x*-axis and specific ^51^Cr-releases are indicated on the *y*-axis. (b) MC2/A2 TCR T cells produce cytokines upon coculture with MC2/A2-positive target cells. T-cell production of IFN*γ* and TNF*α* (in pg/mL) was measured by ELISA in supernatants harvested after an 18 h coculture between T cells and the target cells described in legend to Figure (a) No cytokines were produced by T cells only or Mock T cells cocultured with MC2-positive target cells (data not shown). Measurements were performed in triplicate and expressed as mean values corrected for medium values. Data shown are from representative experiments out of 4 experiments from 2 donors with similar results.

To assess the antigen-specific cytolytic function of MC2/A2 TCR T cells, T cells were cocultured with the MC2/A2-positive tumor cell line, EB81-MEL-2.  Figure 3(a) shows that if these tumor cells were pretreated with IFN*γ*, they were lysed by the TCR T cells. MC2 peptide-loaded HLA-A2 positive B cells (BSM) were lysed very efficiently, whereas gp100 peptide-loaded B cells were not recognized. Additionally, TCR T cells produced IFN*γ* but not TNF*α* in response to IFN*γ* pretreated EB81-MEL-2 cells, although T cells produced both IFN*γ* and TNF*α* in response to MC2 peptide-loaded cells (Figure 3(b)). No IFN*γ* was produced by MC2/A2 TCR T cells in response to MC2^pos^/A2^neg^ tumor cells (*Supplementary Figure  1(b)*).

### 3.4. TCR-V*α*38/V*β*2 Chains Provide T Cells with the Ability to Bind MA3_243–258_/HLA-DP4 Ligands

**Figure 4:**
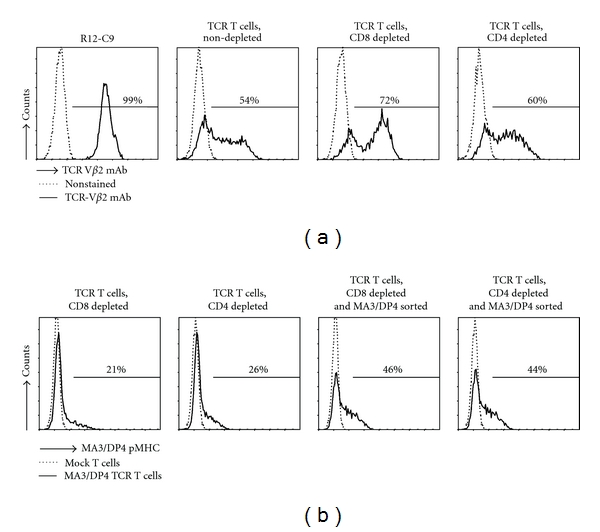
Surface expression of MA3/DP4 TCR on human primary T cells following gene transfer. Human primary T cells transduced with MA3/DP4 TCR*αβ* genes were stained with TCR-V*β*2 mAb (in which case nonstained MA3/DP4 TCR T cells served as a negative control since control TCR*αβ* genes also comprise the TCR-V*β*2 chain) (a) or MA3/DP4 tetramer (b) prior to analysis by flow cytometry. In (a), the following T cells were analyzed: parental CD4 T-cell clone R12-C9; TCR T cells, nondepleted (bulk) and TCR T cells depleted for either CD8 or CD4 T cells. These T-cell populations are not FAC sorted. In (b), TCR-transduced T cells, depleted for either CD8 or CD4 T cells nonsorted, or FAC sorted with MA3/DP4 tetramer, were analyzed. Results are from a representative transduction out of 4 transductions of PBMC from 2 donors with similar results.

MA3/DP4 TCR-transduced T cells, whether depleted or not for either the CD4 or CD8 T-cell subset, expressed high levels of TCR-V*β*2 (Figure 4(a)). Sorting the T cells after gene transfer for high-pMHC binding resulted in expressions that improved by a factor of two (21 and 26% pMHC binding prior to sorting versus 48 and 44% post sorting for CD4 and CD8-depleted T cells, resp.) (see Figure 4(b)). Mock T cells did not bind MA3/DP4 pMHC multimers. Similar to the MC2/A2 TCR, MA3/DP4 TCR expression was stable for at least three months (data not shown).

### 3.5. MA3/DP4 TCR T Cells Recognize Antigen-Positive B Cells but Not Melanoma Cells Natively Expressing MA3 Antigen

**Figure 5:**
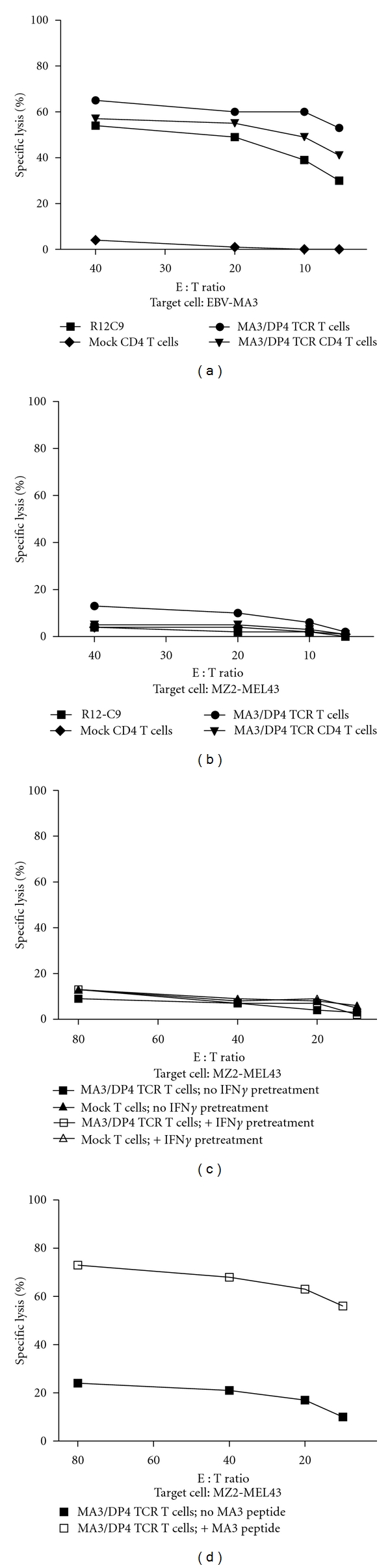
MA3/DP4 TCR T cells specifically lyse MA3-transduced or peptide-loaded B cells, but not MA3-positive melanoma cells. (a) MA3/DP4 TCR T cells specifically lyse DP4-positive B cells transduced with MA3-encoding cDNA. Human T cells were tested in a 6 h ^51^Cr-release assay using EBV-MA3 target cells. The following effector T cells were used: CD4 T-cell clone R12-C9, MA3/DP4 TCR T cells, nondepleted T cells, MA3/DP4 TCR T cells depleted for CD8 T cells, or Mock T cells depleted for CD8 T cells. MA3-negative, DP4-positive B cells (BSM) were not recognized by MA3/DP4 TCR T cells (data not shown). (b) MA3/DP4 TCR T cells do not lyse MZ2-MEL43 melanoma cells, natively expressing MA3 and DP4. Effector T cells used were those described in legend to Figure (a). (c) MA3/DP4 TCR T cells do not lyse MZ2-MEL43 melanoma cells that are pretreated with IFN*γ*. Target cells were MZ2-MEL43 cells that were either pretreated with IFN*γ* or not, and effector T cells were MA3/DP4 TCR or Mock T cells. (d) MA3/DP4 TCR T cells lyse MZ2-MEL43 melanoma cells that are pulsed with MA3 peptide. Target cells were MZ2-MEL43 cells that were either pulsed with MA3 peptide or not, and effector T cells were MA3/DP4 TCR T cells. Measurements were performed in triplicate and expressed as mean values corrected for medium values. Data are from representative experiments out of 3 experiments with similar results.

**Figure 6:**
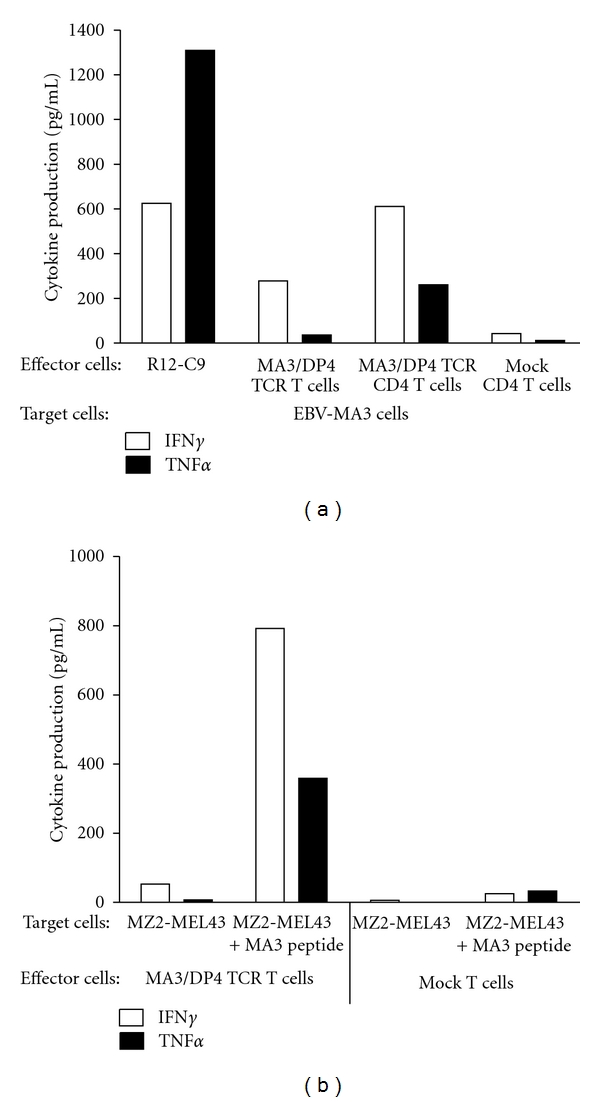
MA3/DP4 TCR T cells specifically produce IFN*γ* and TNF*α* upon coculture with MA3-transduced or peptide-loaded B cells, but not MA3-positive melanoma cells. Cytokine production is determined in supernatants of T cells after an 18 h co-culture with (a) DP4-positive B cells transduced with Ii-MA3 cDNA (EBV-MA3) or (b) MZ2-MEL43 cells loaded with MA3 peptide or not. In (a), effector T cells were: the CD4 T-cell clone R12-C9; MA3/DP4 TCR or Mock T cells, either nondepleted or depleted for CD8 T cells. MA3-negative, DP4-positive B cells (such BSM) were not recognized by MA3/DP4 TCR T cells (data not shown). In (b), MA3/DP4 TCR or Mock T cells, non-depleted, were used as effector T cells. Supernatants were harvested and analyzed for IFN*γ* and TNF*α* by ELISA. Measurements were performed in triplicate and expressed as mean values corrected for medium values. Data are from representative experiments out of 3 experiments with similar results.

MA3/DP4 TCR T cells specifically lysed MA3/DP4 positive EBV B cells (EBV-MA3) (Figure 5(a)). Depleting MA3/DP4 TCR T cells for CD8 T cells resulted in CD4 T cells with a cytotoxic capacity similar to that of nondepleted T cells (mainly being of the CD8 T-cell subset). T cells transduced with the TCR*β* chain of the MA3/DP4 TCR and a TCR*α* chain from a human spleen cDNA library served as a negative control (referred to as Mock T cells) and did not lyse MA3-positive B cells (Figure 5(a)). MA3/DP4 TCR T cells did not lyse MZ2-MEL43 melanoma cells, which naturally express the MA3/DP4 antigen (Figure 5(b)). Pretreatment with IFN*γ* did not, but addition of MA3 peptide did, enhance killing of the MZ2-MEL43 melanoma cells by MA3/DP4 TCR T cells (Figures 5(c) and 5(d)). Next, we showed that MA3/DP4 TCR T cells, but not Mock T cells, produced IFN*γ* and to a lesser extent TNF*α* in response to EBV-MA3 cells, with CD4 T cells as the predominant source of both cytokines (Figure 6(a)). It is noteworthy that TCR CD4 T cells, but not the parental R12-C9 T-cell clone, produced more IFN*γ* than TNF*α* (Figure 6(a)). Responses of TCR T cells towards EBV-MA3 B cells were blocked with an anti-TCR V*β*2 antibody, whereas those towards MA3^pos^/DP4^neg^ tumor cells (*Supplementary Figure  2(b)*) and MA3^neg^/DP4^pos^ B cells were always negative (data not shown). T cells expressing MA3/DP4 TCR (but not Mock) were able to respond to MZ2-MEL43 melanoma cells only when target cells were preloaded with MA3 16 mer peptide; this demonstrates that these melanoma cells can be sensitized to peptide-specific T-cell functions (i.e., cytotoxicity: data not shown; production of IFN*γ* and TNF*α*: Figure 6(b)).

### 3.6. MA3/DP4 TCR CD4 T Cells Produce Cytokines upon Coculture with MA3-Loaded Autologous Monocyte-Derived DC

**Figure 7:**
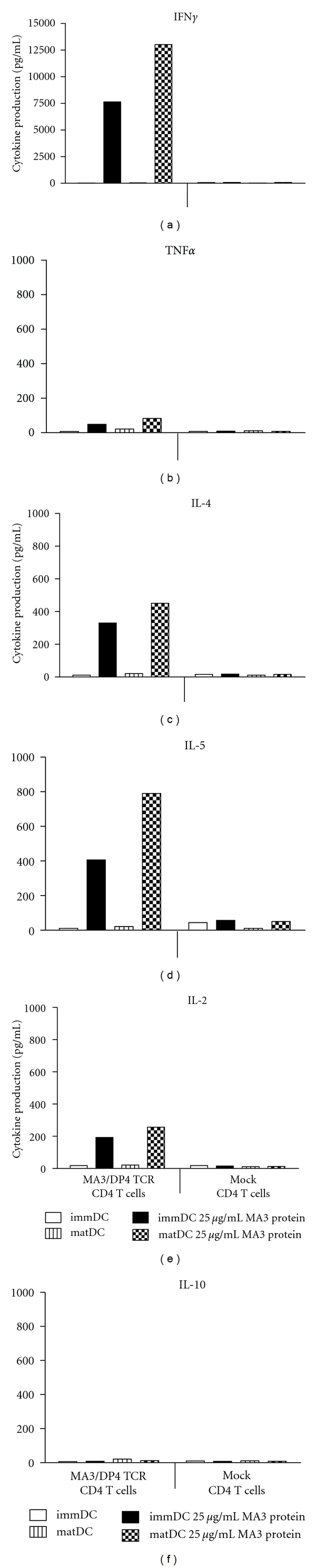
MA3/DP4 TCR CD4 T cells produce cytokines upon coculture with MA3 protein-loaded autologous dendritic cells. MA3/DP4 TCR, and Mock CD4 T cells were cultured with immature or mature autologous dendritic cells that were either loaded with 25 *μ*g/mL MA3 protein or not. After 4 days, supernatants were harvested and analyzed for cytokine production by cytokine bead arrays. Cytokine production was not detected in case T cells were cultured without dendritic cells (data not shown). Measurements were performed in duplicate and expressed as mean values. Data are from a representative experiment out of 2 experiments with similar results.

Since MA3/DP4 TCR T cells are unable to directly recognize antigen-positive melanoma cells, an ability that is generally expected only for antitumor CD8 T cells, we analyzed a more typical CD4 T-cell response that is based on (cross-) presentation of tumor antigens by DC. To this end, MA3/DP4 TCR and Mock CD4 T cells were cocultured with immature or mature DC derived from autologous monocytes using two different MA3 protein concentrations for DC uptake. After 4 days, the production of various cytokines was determined in supernatants of the DC: T-cell cocultures. Upon coculture with the MA3-protein-loaded DC, MA3/DP4 TCR CD4 T cells (but not Mock CD4 T cells) produced significant amounts of IFN*γ* (up to 1300 pg/mL) and to a lesser extent TNF*α*, IL-2, IL-4, and IL-5 (Figure 7). MA3-specific production of IL-10 was negligible. T-cell cytokine production was negligible or absent when either nonprotein-loaded immature or mature DC or Mock T cells were used.

## 4. Discussion

Redirection of T cells towards tumor-specific yet clinically safe antigens holds great promise for the treatment of melanoma and other tumor types. In the current paper, we have studied MAGE-C2/HLA-A2 (MC2/A2) and MAGE-A3/HLA-DP4 (MA3/DP4) as targets of TCR T cells; besides being prevalent in the patient population, these antigens are uniquely expressed by tumors and have proven value in initiating clinically effective CD8 and CD4 T-cell responses [23, 27, 28, 46].

MC2/A2 and MA3/DP4 TCR*αβ* genes were derived from T-cell clones obtained from MAGE-vaccinated patients and were subsequently characterized following TCR gene transfer (see Figure 1). The CD8 T-cell clone EB81-CTL 16 expressed the MC2/A2-specific TCR-V*α*3 and TCR-V*β*28 chains. Upon gene transfer, primary human T cells bound pMHC and demonstrated MC2-specific T-cell functions. TCR T cells killed and produced IFN*γ* and TNF*α* upon coculture not only with MC2 peptide-pulsed HLA-A2-positive target cells but also native MC2-positive, HLA-A2-positive melanoma cells (see Figures 2 and 3). T-cell responsiveness towards native MC2-positive, HLA-A2-positive melanoma cells (i.e., EB81-MEL-2 cells) was enhanced by IFN*γ* pretreatment, which promotes antigen processing and surface expression of MHC and adhesion molecules. In fact, unlike other antigenic peptides, the MC2 epitope ALKDVEERV (i.e., MC2_336–344_) requires immune proteasomes for proper processing and presentation to T cells [52], supporting the value of MC2/A2 as a target for T-cell therapy. The effective dose of MC2/A2 peptide at which CD8 T cells demonstrate a half-maximal lytic response (i.e., ED50) is 0.75 nM [53]. This value represents a measure of T-cell avidity and lags somewhat behind in comparison to reported values for T cells expressing other MHC class I TCRs (range: 30–100 pM) [54, 55], suggesting a lower-to-intermediate ligand-binding affinity of this TCR. Experiments with mutated pMHC complexes that either prevent or enhance CD8*α* binding (according to [56, 57]; kindly provided by Professor Dr. Andrew Sewell, University of Cardiff, Wales) confirm the CD8-dependency of this MC2 TCR (data not shown).

The CD4 T-cell clone R12-C9 expressed the MA3/DP4 specific TCR-V*α*38 and TCR-V*β*2 chains, which upon gene transfer in primary human T cells, resulted in pMHC binding (see Figure 4). In addition, MA3/DP4 TCR T cells, containing both CD8 and CD4 T cells (i.e., nondepleted), specifically lysed and produced IFN*γ* and TNF*α* upon coculture with B cells either transduced with MA3 antigen (Figures 5(a) and 6(b)) or loaded with MA3 peptide (data not shown). The extent of lysis, a typical measure for CD8 T cell function, was lowered when testing MA3/DP4 TCR CD4 T cells (i.e., depleted for CD8-positive T cells). The responsiveness of MA3/DP4 TCR T cells towards MZ2-MEL43 cells, generally weak and not reproducible, was not enhanced by IFN*γ* pretreatment of target cells, whereas T cells were clearly able to recognize melanoma cells following loading with the MA3 16-mer peptide but not a core 12-mer peptide (TQGFVQENYLEY, i.e., MA3_247–258_) (Figures 5(b), 5(c), 5(d), and 6(b)). Collectively, these data argue that MA3, like other nuclear proteins, may be inefficiently presented by tumor cells, and T-cell responses directed to tumor cells natively expressing MA3, such as those reported for T-cell clones 22 and R12–57 [58, 59] are rare and difficult to reproduce. In fact, when screening a panel of 23 T-cell clones including many patient R12-derived T-cell clones, we were unable to identify a single T-cell clone that responded towards MZ2-MEL43 (data not shown). In this respect, it is noteworthy that R12-derived CD4 T-cell clones show a polyclonal response towards MA3/DP4, with 50% of clonotypes sharing TCR-V*β*12 gene [46]. Thus, MA3/DP4 TCR T cells are able to lyse antigen-positive target cells, but lysis becomes suboptimal in case (low levels of) antigen is presented by tumor cells. The ED50 value of MA3/DP4 peptide in a CD4 T cell IFN*γ* assay is 30 nM [59]. This value is in accordance with reported values for T cells expressing other MHC class II TCRs (range: 40–200 nM) [20, 60]. Notably, functional expressions of MHC class II TCRs, such as reported for NY-ESO-1/DP4 TCR, may depend on the presence of the CD4 coreceptor [20], and are assessed by typical CD4 T cell assays, such as T cell proliferation and cytokine production. In case of MA3/DP4 TCR, we also observed that antigen-specific IFN*γ* production is higher in T cells depleted for CD8 T cells (i.e., CD4 T cells) when compared to nondepleted T cells (i.e., CD4+ CD8 T cells, Figure 6(a)).

Antitumor responses more typical for CD4 T cells are induced by professional antigen-presenting cells, such as DC (reviewed in [61]). DC capture and process tumor antigens and cross-present MHC class II-restricted antigens to CD4 T cells. Following activation, these CD4 T cells provide signals to DC that enhance antigen presentation and costimulation (via cross-linking of CD40) and lead to priming of antigen-specific CD8 CTL function [62]. Importantly, activated CD4 T cells are a major source of IFN*γ*, an effector cytokine with potent tumor regressing activity via inhibition of tumor-induced angiogenesis or activation of tumor-infiltrating macrophages [63–65]. When analyzing DC-induced T-cell responses, we observed significant production of cytokines when immature or mature DC were loaded with MA3 protein and used to stimulate TCR T cells (Figure 7). Decreasing the MA3 protein concentration during maturation of the DC from 25 to 5 *μ*g/mL resulted in only slightly lower but almost comparable cytokine responses (data not shown). These findings extend the observations with two other MHC class II-restricted TCRs specific for human antigens, that is, NY-ESO1/DP4 and DBY/DQ5 [20, 66]. MA3/DP4 TCR-transduced T cells produced high amounts of IFN*γ*, whereas TNF*α*, IL-2, IL-4, and IL-5 were produced to a lesser extent. IL-10 represents the only cytokine with production levels being low (<20 pg/mL) and not different from TCR and Mock-transduced T cells (Figure 7). In addition, coculture with MA3-positive DC resulted in upregulated expression of T-cell activation markers such as CD25 (IL-2R*α* chain) as well as enhanced T-cell proliferation (data not shown). Analysis of DC phenotype and function after coculture with MA3/DP4 TCR T cells was not possible, since DC died at days 1 and 2 after the start of coculture and were completely lost at day 4, which was evidenced by light microscopy and lack of IL-12 production and suggested direct killing of DC by TCR T cells. Our observation that MA3/DP4-specific CD4 T cells recognize MA3-protein-loaded DC rather than MZ2-MEL43 melanoma cells implies that these CD4 T cells yield antitumor activity *in vivo* following cross-presentation by professional antigen presenting cells. The therapeutic benefit of antigen-specific IFN*γ* production have initiated studies in which CD4 T cells were used as recipient T cells for MHC class I-restricted TCR. Not only can CD4 T cells be functionally endowed with MHC I-restricted TCR via gene transfer [19, 67, 68], but also can genetic cointroduction of CD8*α* skew TCR-engineered T cells towards an antigen-specific Th1 type T-cell response [69]. Vice versa, the introduction of a MHC class II TCR and CD4 coreceptor in CD8 T cells may lead to the generation of T cells with combined helper and effector T-cell functions [66].

In extension to our results with wild type MC2 and MA3 TCRs, we have tested gene optimization, a transgene cassette and another retroviral vector to enhance functional expression of TCR transgenes [70–72]. To this end, we have cloned codon-optimized MC2 and MA3 TCRs in TCR*β*-2A-TCR*α* cassette-containing pMP71 vectors and demonstrated significant TCR surface expression and MAGE-specific IFN*γ* production by CD3 mAb-activated and transduced PBMC (note that results with pMP71: optTCR*β*-2A-TCR*α* reflect bulk, nonsorted T cells, *Supplementary Figures  1 and 2*). In preparation of clinical studies, we propose the following additional strategies to enhance the therapeutic efficacy of T cells gene engineered with MC2 and MA3 TCRs. *First*, administration of common *γ*-cytokines, such as a combination of IL-15 and IL-21, to cultures of TCR T cells prior to patient infusion will yield T cells that show limited T-cell differentiation, and are better equipped to persist and function *in vivo* ([73]; Lamers, manuscript in preparation). *Second*, we propose preconditioning of patients that, apart from nonmyeloablative treatment with cyclophosphamide and fludarabine [1], includes treatment with the DNA hypomethylating agent 5-AZA-CdR. Such treatment, already used clinically, is reported to enhance expression of MAGE antigens and HLA in melanoma [26, 74]. And *third*, cotreatment with MC2 TCR-transduced CD8 and MA3 TCR CD4 T cells may be of particular interest to boost antitumor immunity and counteract selected growth of epitope-negative tumor variants. In fact, we have recently demonstrated that single-epitope targeting of melanoma by TCR-engineered T cells results in highly effective but transient regression in HLA-A2 transgenic mice and that more effective strategies likely require multi-epitope targeting (Straetemans, manuscript submitted). The proposed dual-epitope targeting approach may prove especially effective for CTA epitopes because of their coregulated expression pattern in tumor cells, with the vast majority of tumor cells expressing two or more CTAs [75]. Testing of cell lines derived from tumors other than melanoma has started in our laboratory, and may provide a preclinical rationale to extend the proposed treatment to nonmelanoma tumors.

In short, we have cloned and *in vitro* validated two MAGE-specific TCRs that warrant clinical testing in TCR gene therapy in melanoma patients and in other patients with cancers expressing the MC2 and MA3 antigens.

## Supplementary Material

Online Supplementary Material includes the description, expression and the function of the optimized MC2 and MA3 TCRs. Figure S1 shows the expression and function of the optimized MC2/A2 TCR and figure S2 shows the expression and function of the MA3/DP4 TCR.
