# Supplementary material for: TCR Gene Transfer: MAGE-C2/HLA-A2 and MAGE-A3/HLA-DP4 Epitopes as Melanoma-Specific Immune Targets
Source: Clin Dev Immunol. 2012 Feb 12;2012:586314. doi: 10.1155/2012/586314 (PMC3287115; doi:10.1155/2012/586314)
Supplement: Supplementary file 1 — Online Supplementary Material includes the description, expression and the function of the optimized MC2 and MA3 TCRs. Figure S1 shows the expression and function of the optimized MC2/A2 TCR and figure S2 shows the expression and function of the MA3/DP4 TCR. [file 586314.f1.pdf]

Supplementary text and figures

<u>Additional tumor cell lines</u>

Breast carcinoma cell line EVSA-T ($MC2^{pos}/A2^{neg}$) and esophagus carcinoma cell line TE-4 ($MA3^{pos}/DP4^{neg}$) served as negative control cell lines in T cell assays, and were cultured with DMEM with 10% Fetal Bovine Serum, glutamine, antibiotics and 1% MEM non-essential amino acids.

Additional TCR constructs

MC2/A2 and MA3/DP4 TCRα and β genes were codon optimized (GeneArt, Regensburg, Germany) and cloned into the pMP71 vector (kindly provided by prof. Wolfgang Uckert, Max-Delbrück Center, Berlin, Germany), with TCRβ and TCRα genes separated by an optimized T2A ribosome skipping sequence (abbreviated as pMP71:opt TCRβ-T2A-α). TCRs were introduced into T cells and analyzed for surface expression, pMHC binding and TCR-mediated IFNγ production (*Supplementary Figures 1 and 2*). MC2/A2 TCR surface expression was measured with a FITC-labeled anti-TCRVβ28 mAb (clone CH92, Beckman Coulter) or MC2/A2 PE-labeled pMHC tetramers in combination with either anti-CD3 mAb or a combination of anti-CD3 and CD8 mAbs. MA3/DP4 surface expression was measured with a PE-labeled anti-TCRVβ2 mAb or MA3/DP4 PE-labeled pMHC tetramer in combination with either anti-CD3 mAb or a combination of anti-CD3 and CD4 mAbs.

Figure S1

A

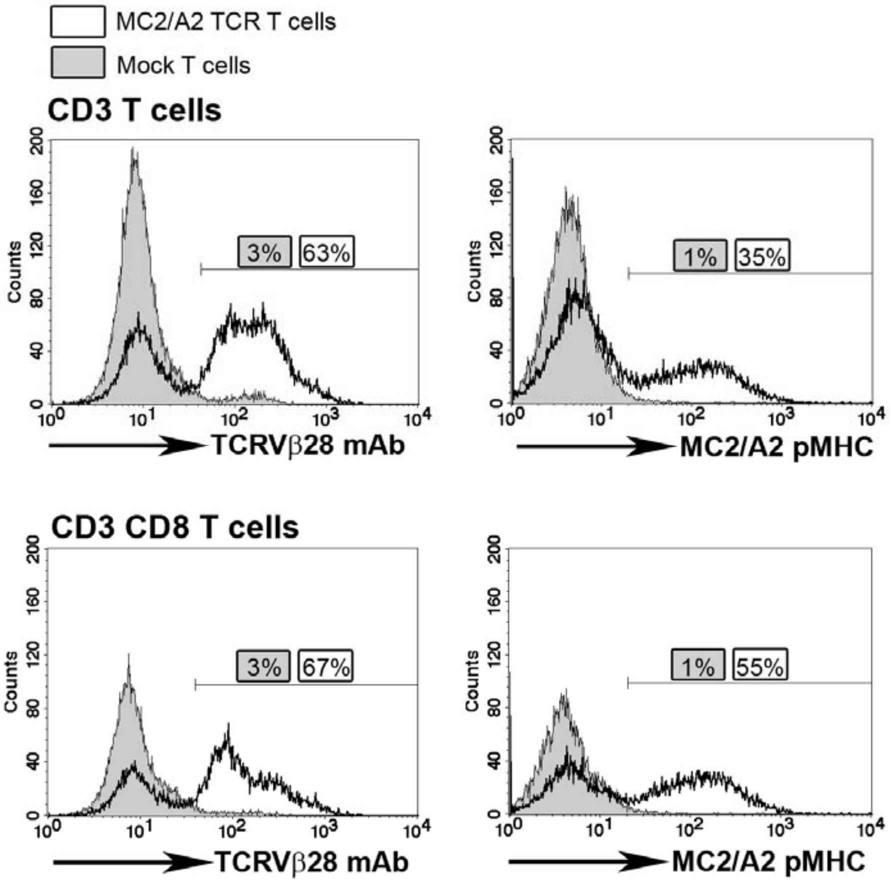

MC2/A2 TCR T cells
Mock T cells
CD3 T cells
3%
63%
1%
35%
Counts
TCRVβ28 mAb
MC2/A2 pMHC
CD3 CD8 T cells
3%
67%
1%
55%

B

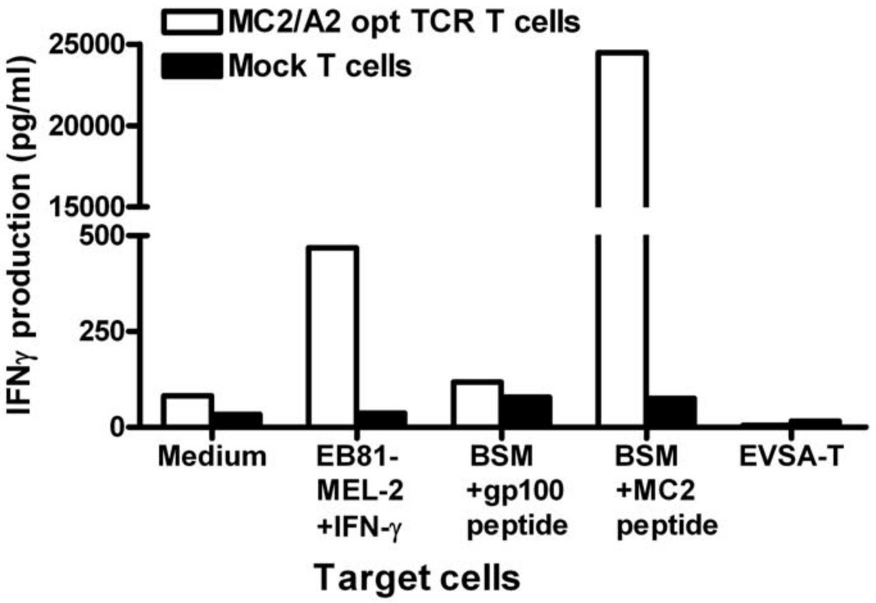

MC2/A2 opt TCR T cells
Mock T cells
IFNγ production (pg/ml)
25000
20000
15000
500
250
0
Medium
EB81-MEL-2 +IFN-γ
BSM +gp100 peptide
BSM +MC2 peptide
EVSA-T
Target cells

Figure S1. Codon-optimized MC2 TCR genes are functionally expressed in T cells. (A) Surface expression of codon-optimized MC2 TCR genes in PBMC. PBMCs were activated by anti-CD3 mAb and transduced with pMP71:opt MC2/A2 TCRβ-2A-α or control genes (i.e., pSTITCH: CAIX CAR:CD4γ (1), termed Mock T cells). CD3 and CD3, CD8-positive T cells were analyzed for TCRVβ28 expression and MC2/A2 pMHC binding by flow cytometry. **(B)** MC2/A2-specific IFNγ production by PBMC transduced with codon-optimized MC2 TCR genes. Target cells were: MC2-positive, A2-positive EB81-MEL-2 melanoma cells; **$MC2^{pos}/A2^{neg}$ EVSA-T breast carcinoma cells,** both pre-treated with IFNγ; or BSM B cells loaded with gp100 or MC2 peptide. Effector T cells were MC2/A2 TCR or Mock T cells (as in Figure S1A). T cells only were included as an additional control. Supernatants were harvested after 20 h co-culture between effector T cells and target cells, and analyzed for the presence of IFNγ by ELISA. Data are from representative experiments out of 15 experiments from 5 donors with similar results.

Figure S2

A

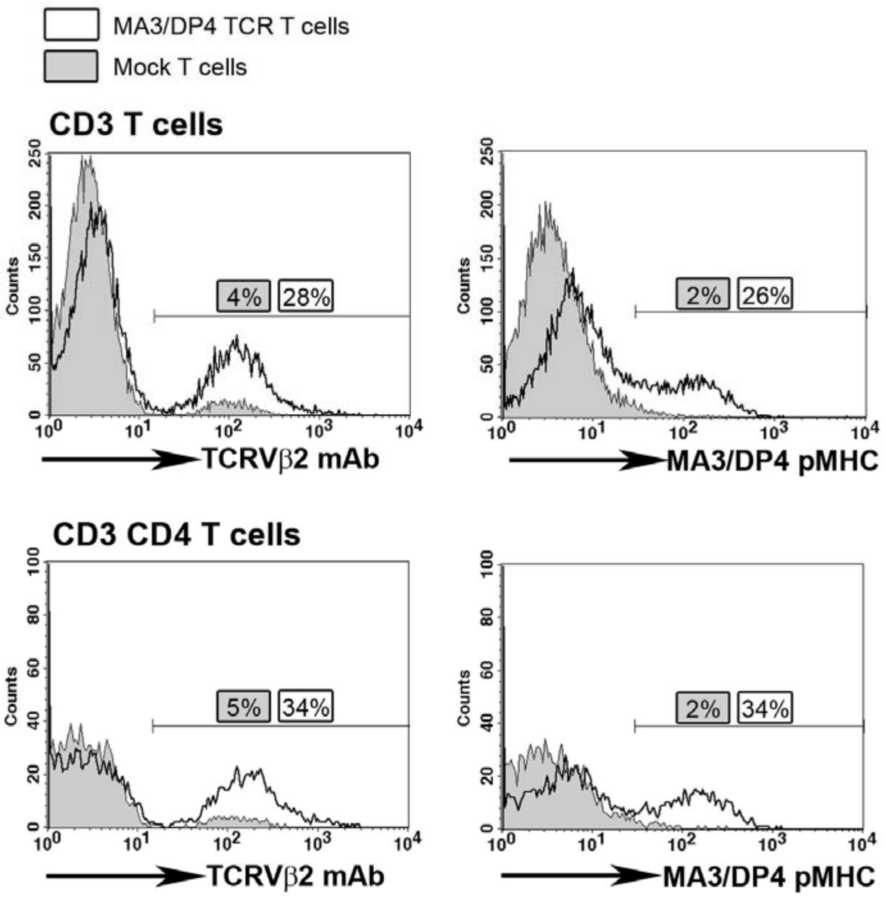

MA3/DP4 TCR T cells
Mock T cells
CD3 T cells
CD3 CD4 T cells
Counts
4%
28%
2%
26%
5%
34%
2%
34%
TCRVβ2 mAb
MA3/DP4 pMHC

B

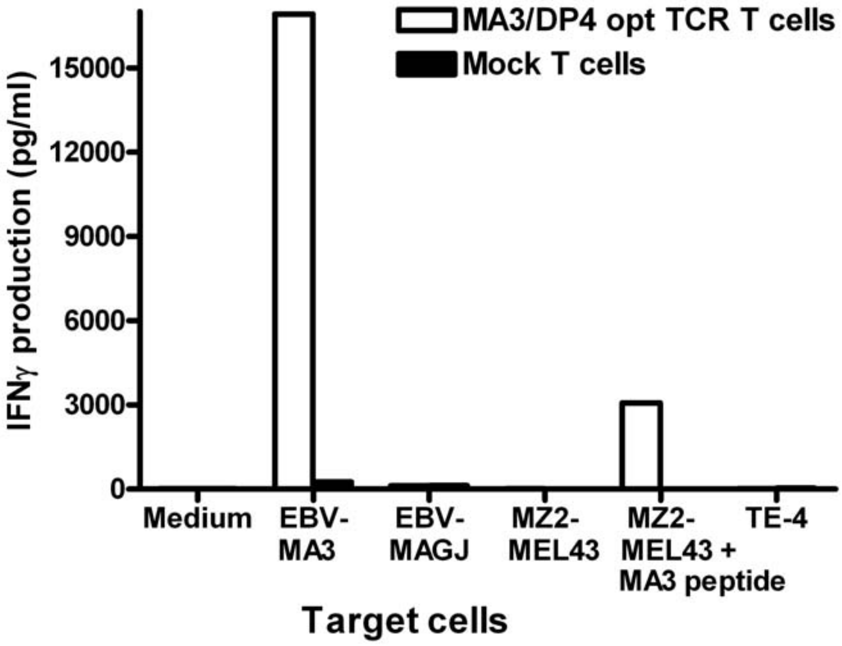

MA3/DP4 opt TCR T cells
Mock T cells
IFNγ production (pg/ml)
15000
12000
9000
6000
3000
0
Medium
EBV-MA3
EBV-MAGJ
MZ2-MEL43
MZ2-MEL43 + MA3 peptide
TE-4
Target cells

Figure S2. Codon-optimized MA3 TCR genes are functionally expressed in T cells. (A) Surface expression of codon-optimized MA3 TCR genes in PBMC. PBMCs were activated by anti-CD3 mAb and transduced with pMP71:opt MA3/DP4 TCRβ-2A-α or control genes (i.e., pMP71:opt MC2/A2 TCRβ-2A-α, termed Mock T cells). CD3 and CD3, CD4-positive T cells were analyzed for TCRVβ22 expression and MA3/DP4 pMHC binding by flow cytometry. **(B)** MA3/DP4-specific IFNγ production by PBMC transduced with codon-optimized MA3 TCR genes. Target cells were: MA3-positive B cells (EBV-MA3); MA3-negative B cells (EBV-MAGJ); MZ2-MEL43 melanoma cells loaded with MA3 peptide or not (all HLA-DP4-positive cells) and **$MA3^{pos}/DP4^{neg}$ TE-4 esophagus carcinoma cells**. Effector T cells were MA3/DP4 TCR or Mock T cells (as in Figure S2A). T cells only were included as an additional control. Supernatants were harvested after a 20 h co-culture between effector T cells and target cells, and analyzed for the presence of IFNγ by ELISA. Data are from representative experiments out of 6 experiments from 2 donors with similar results.

References

1. Lamers CH, Sleijfer S, Vulto AG, *et al.* Treatment of metastatic renal cell carcinoma with autologous T-lymphocytes genetically retargeted against carbonic anhydrase IX: first clinical experience. J Clin Oncol 2006;24(13):e20-2.
